# Determinants of the calibration of SAPS II and SAPS 3 mortality scores in intensive care: a European multicenter study

**DOI:** 10.1186/s13054-017-1673-6

**Published:** 2017-04-04

**Authors:** Antoine Poncet, Thomas V. Perneger, Paolo Merlani, Maurizia Capuzzo, Christophe Combescure

**Affiliations:** 1 Clinical Research Center, Faculty of Medicine, University of Geneva, Geneva 4, 1211 Geneva, Switzerland; 2 Division of clinical epidemiology, Department of health and community medicine, University Hospitals of Geneva, Rue Gabrielle Perret-Gentil 4, 1211 Geneva, Switzerland; 3 Department of Anesthesiology, Intensive Care and Pharmacology, University Hospitals of Geneva, Rue Gabrielle Perret-Gentil 4, 1211 Geneva, Switzerland; 4 Intensive Care Unit, Lugano Regional Hospital, Via Tesserete 46, 6900 Lugano, Switzerland; 5 Department of Morphology, Surgery and Experimental Medicine, Section of Anesthesia and Intensive Care, Sant’Anna Hospital, University of Ferrara, Via Aldo Moro 8, Cona, 44124 Ferrara, Italy

**Keywords:** Calibration, SAPS II, SAPS 3, Determinants

## Abstract

**Background:**

The aim of the Simplified Acute Physiology Score (SAPS) II and SAPS 3 is to predict the mortality of patients admitted to intensive care units (ICUs). Previous studies have suggested that the calibration of these scores may vary across countries, centers, and/or characteristics of patients. In the present study, we aimed to assess determinants of the calibration of these scores.

**Methods:**

We assessed the calibration of the SAPS II and SAPS 3 scores among 5266 patients admitted to ICUs during a 4-week period at 120 centers in 17 European countries. We obtained calibration curves, Brier scores, and standardized mortality ratios. Points attributed to SAPS items were reevaluated and compared with those of the original scores. Finally, we tested associations between the calibration and center characteristics.

**Results:**

The mortality was overestimated by both scores: The standardized mortality ratios were 0.75 (95% CI 0.71–0.79) for the SAPS II score and 0.91 (95% CI 0.86–0.96) for the SAPS 3 score. This overestimation was partially explained by changes in associations between some items of the scores and mortality, especially the heart rate, Glasgow Coma Scale score, and diagnosis of AIDS for SAPS II. The calibration of both scores was better in countries with low health expenditures. The between-center variability in calibration curves was much greater than expected by chance.

**Conclusions:**

Both scores overestimate current mortality among European ICU patients. The magnitude of the miscalibration of SAPS II and SAPS 3 scores depends not only on patient characteristics but also on center characteristics. Furthermore, much between-center variability in calibration remains unexplained by these factors.

**Trial registration:**

ClinicalTrials.gov identifier: NCT01422070. Registered 19 August 2011.

**Electronic supplementary material:**

The online version of this article (doi:10.1186/s13054-017-1673-6) contains supplementary material, which is available to authorized users.

## Background

Scores that predict in-hospital survival of patients admitted to the intensive care unit (ICU) can be used for the assessment of ICU performance [1–4], to measure patient case mix, and to make statistical adjustments for between-group comparisons. Several predictive scores have been developed for this purpose, including the Simplified Acute Physiology Score (SAPS) II and SAPS 3 [5, 6].

Desirable characteristics of predictive scores are the capacity to distinguish between patients who will experience the studied outcome and patients who will not (i.e., discrimination) and the agreement between the observed occurrence of the outcome and the risk predicted by the score (i.e., calibration) [7]. If the discrimination is poor, the predictive score is useless in clinical practice, and calibration is irrelevant. When the discrimination is acceptable, it is necessary to investigate the quality of the calibration. Researchers in various studies have assessed the calibration of the SAPS II and SAPS 3 scores and, on the whole, found a poor calibration in European countries, especially for SAPS II. Whereas some researchers have reported that the SAPS II overestimated mortality [8–10], others have found the opposite [4, 11, 12]. The calibration of predictive scores can change over time because ICU populations change and new diagnostic, therapeutic and prognostic techniques become available [3]. Additionally, calibration of scores can vary across countries and even between centers within a country. Villers et al. reported a high level of heterogeneity in calibration of the SAPS II between French centers [12]. Indeed, it is possible that the reasons for admission to an ICU differ between centers, such that risk factors for mortality that are important in one center will not be useful in another, thus reducing discriminative ability. It is also possible that the general level of care differs between centers, which would influence the background risk of dying and therefore affect the calibration of the score [13]. Ethical issues such as limitation or withdrawal of therapies can also change between geographic regions and probably between centers [14].

In this study, we assessed the calibration of the SAPS II and SAPS 3 in patients admitted to ICUs in 17 European countries and sought to identify sources of miscalibration. We hypothesized that the magnitude of the association between some items of the scores and death might have decreased since the development of the scores, especially for the SAPS II, which was developed 20 years ago. We reevaluated points attributed to SAPS items and compared them with those of the original scores. We investigated the impact of the modification of scoring on calibration curves. In addition, we explored whether characteristics of centers contributed to miscalibration.

## Methods

### ELOISE study and subset of analyzed data

The primary objective of the European Mortality & Length of Intensive Care Unit Stay Evaluation (ELOISE) study was to estimate the effect on hospital mortality of the presence of an intermediate care unit (IMCU) in the hospital [15]. The analysis presented in this paper is an ancillary study. The ELOISE study included 5834 patients admitted during one of two 4-week periods (either in November 2011 or in February 2012) to 167 ICUs from 17 European countries. Excluding from our analysis ICUs that recruited fewer than 20 patients for the ELOISE study, so as to have enough observations to estimate a calibration curve for each center and enough centers to explore heterogeneity, we analyzed data of 5266 patients from 120 centers located in 17 countries. Data collection is detailed in Additional file 1.

### Calculation of SAPS II and SAPS 3 scores

The scores and the predicted mortality were calculated following the original equations for both SAPS scores [5, 6]. The risk predicted by the SAPS 3 score was assessed with equations customized for geographical area (Central/Western, Eastern, Northern, and Southern Europe) [6].

### Assessment of calibration of SAPS II and SAPS 3 scores

The calibration curves of the SAPS II and SAPS 3 scores for the prediction of in-hospital death were obtained to show the relationship between the observed and the predicted mortality. The observed risk function of the predicted mortality was assessed using smooth kernel functions [16] and was plotted against the predicted mortality. The identity line represents a perfect calibration of the score. If the curve is below (above) the identity line, the score overestimates (underestimates) the mortality. The greater the deviation from the identity line, the greater the miscalibration. Additionally, we calculated the Brier score and the standardized mortality ratio (SMR) of the scores [7]. The Brier score is the mean squared difference between the probability of death and the actual outcome (0 if the patient survives, 1 if the patient dies); a smaller value is better [17]. An SMR greater (or lower) than 1 indicates an underestimation (or overestimation) of the mortality by the predictive score.

### Calibration and patient characteristics

We reassessed the points attributed to each item in the SAPS II following the methodology used in the original work [5]. The associations between the components and mortality were based on a multivariable logistic regression model, and the number of points of an item were the nearest integer of ten times the estimated regression coefficient. If the associations obtained with data from the ELOISE study changed from the original work, the number of attributed points would also change. A greater difference between original and attributed points reflects a greater impact on calibration. Similar analyses were conducted for the SAPS 3, but using a logistic regression model with mixed effects (with patients’ characteristics as fixed effects and centers as random effects on the intercept) to reproduce the methodology followed in the in the original work [6]. A post hoc analysis was conducted to assess the calibration curves, the SMRs, and the Brier scores according to the reasons for admission to the ICU. Only reasons with more than 200 admissions were investigated (cardiovascular reason, digestive reason, neurological reason, respiratory reason, severe trauma, basic observation).

### Calibration and center characteristics

We also hypothesized that some centers’ characteristics may influence the calibration. First, we verified whether the variability in the calibration across centers is compatible with the variability caused by random sampling. For this purpose, we fitted a calibration curve for each of the 120 centers. The variances of the center-specific Brier scores and SMRs reflected the between-center variability in calibration. A permutation test was conducted to determine if the observed value of these variances was compatible with the hypothesis that the calibration is the same for all centers. The permutation test consisted in attributing patients at random to centers, computing their Brier scores and SMRs, then obtaining the variances of these quantities, and repeating this procedure 1000 times. The resulting distribution of the variances of Brier scores and SRMs reflects between-center variance that is attributable only to chance; the actual observed values were compared with these distributions. To evaluate if center characteristics have an effect on the calibration of the SAPS II score, we modeled the calibration curve using the approach proposed by Finazzi et al., and we introduced interaction terms between the centers’ characteristics and the logit values of the predicted mortality [18]. This analysis was conducted for each of the following characteristics: 2012 national health expenditure in percentage of gross domestic product (GDP), number of hospital beds (<500, 500–1000, >1000 beds), presence of an IMCU, presence of IMCU beds inside the ICU, number of ICU adjusted beds (two IMCU beds inside the ICU equal one ICU bed [15]), possibility of allocating additional beds inside the ICU, and the nurse/patient daytime ratio (<0.5, 0.5–1, >1). The same analyses were conducted for the SAPS 3 score.

Statistical methods are detailed in Additional file 2. All statistical analyses were performed with the R statistical software package (https://www.r-project.org/; R Foundation for Statistical Computing, Vienna, Austria). The significance level was set at 0.05, and all statistical tests were two-sided.

## Results

The characteristics of the 120 participating centers and 5266 participating patients are described in Table 1. Most hospitals had a capacity of 500–1000 beds, were located in countries with annual health expenditures greater than 8% of GPD, had an IMCU, had a daytime nurse/patient ratio between 0.5 and 1, and had a number of ICU adjusted beds greater than 12. Patients were 62.4 years old, on average (range 18–98), at ICU admission, and 60% were men. Admissions to the ICU were unplanned for 69% of patients, and 49% were admitted following surgery.

**Table 1:** Centers and patients characteristics

Center characteristics	Centers (*n* = 120)	Patients (*n* = 5266)
Number of patients/ICU, median [range]	32 [20–89]	
Number of hospital beds^a^, *n* (%)		
< 500	39 (33.6%)	1403 (27.5%)
500–1000	54 (46.6%)	2630 (51.5%)
> 1000	23 (19.8%)	1072 (21.0%)
Health expenditure (% of GDP^b^), *n* (%)		
< 8%	19 (15.8%)	961 (18.2%)
8% to 10%	51 (42.5%)	2107 (40.0%)
> 10%	50 (41.7%)	2198 (41.7%)
IMCU (intermediate care unit), *n* (%)		
Yes	103 (85.8%)	4563 (86.7%)
Daytime nurse/patient ratio, *n* (%)		
< 0.5	25 (20.8%)	1150 (21.8%)
0.5–1	58 (48.3%)	2536 (48.2%)
> 1	37 (30.8%)	1580 (30.0%)
ICU adjusted beds, *n* (%)		
< 8	19 (15.8%)	571 (10.8%)
8–12	49 (40.8%)	1901 (36.1%)
> 12	52 (43.3%)	2794 (53.1%)
Possibility of extra beds inside ICU, *n* (%)		
Yes	24 (20.0%)	1114 (21.2%)
Patient characteristics		
Male sex, *n* (%)		3143 (59.7%)
Age, years, mean ± SD		62.4 ± 16.9
SAPS II^c^, mean ± SD		
Score		39.3 ± 21.3
Predicted mortality		30.1% ± 30.2
SAPS 3^d^, mean ± SD		
Score		35.0 ± 17.2
Predicted mortality		25.4% ± 24.5
Hospital mortality, *n* (%)		
Death		1194 (22.7%)
ICU admission, *n* (%)		
Unplanned		3613 (68.7%)
Surgery, *n* (%)		
Emergency surgery		983 (18.7%)
No surgery		2663 (50.6%)
Scheduled surgery		1612 (30.7%)
Reason for admission^e^, *n* (%)		
Basic observation		1111 (21.1%)
Cardiovascular		1252 (23.8%)
Digestive		526 (10.0%)
Hematological		77 (1.5%)
Hepatic		62 (1.2%)
Metabolic		195 (3.7%)
Neurological		800 (15.2%)
Renal		200 (3.8%)
Respiratory		980 (18.6%)
Severe trauma		255 (4.8%)

*Abbreviations: GDP* Gross domestic product, *ICU* Intensive care unit, *IMCU* Intermediate care unit, *SAPS* Simplified Acute Physiology Score

^a^The total number of hospitals giving information on the number of hospital beds was 116

^b^Health expenditure in the country of the center expressed in percentage of GDP. (*Source*: World Bank [http://data.worldbank.org/indicator/SH.XPD.TOTL.ZS].)

^c^The medians (interquartile ranges) were 35 [23–52] for the SAPS II score and 16.7% [5.2% to 50.7%] for the mortality predicted by the SAPS II score

^d^The medians (interquartile ranges) were 33 [22–46] for the SAPS 3 score and 15.9% [5.1% to 39.8%] for the mortality predicted by the SAPS 3 score

^e^Reasons for admission were not exclusive (except “basic observation,” which is exclusive of all other reasons)

### Calibration of SAPS II and SAPS 3 scores

**Fig. 1:**
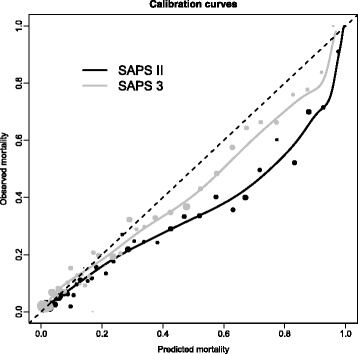
Calibration curves for the Simplified Acute Physiology Score (SAPS) II (*dark line*) and the SAPS 3 (*gray line*) obtained with a kernel function. The calibration curve represents the relationship between the mortality predicted by the score (*x*-axis) and the observed mortality (*y*-axis). The identity line (*dashed line*) represents a perfect calibration. A calibration curve below the identity line indicates that the score overestimates the mortality. The *black* and *gray circles* represent the estimates of the observed mortality in sample, stratified by levels of predicted mortality (by step of 0.01 up to a predicted mortality of 0.20, by step of 0.025 for a predicted mortality from 0.20 to 0.35, and by step of 0.05 for a predicted mortality greater than 0.35). The size of the *circles* is proportional to the number of patients in categories of predicted mortality

The SAPS II and SAPS 3 scores were collected for 5209 (98.9%) and 5206 (98.9%) patients. The number of deaths expected by the SAPS II score was 1568 (30.1%), whereas the number of observed deaths was 1194 (22.7%), resulting in an SMR of 0.75 (95% CI 0.71–0.79). The calibration curve (Fig. 1) below the identity line confirmed that the SAPS II score globally overestimated the mortality. The magnitude of the overestimation varied with the level of the mortality predicted by the SAPS II score. The predicted mortality was reasonably accurate for low-risk patients: the overestimation was less than 0.04 up to a predicted mortality of 0.20. The overestimation became important for patients with intermediate and high levels of predicted mortality (between 0.50 and 0.90): The overestimation reached 0.25 for a predicted mortality around 0.75. The Brier score for the prediction by SAPS II was 0.132 (95% CI 0.127–0.137). If the score was not able to discriminate between deceased patients and survivors (i.e., if the observed risk of death of 0.227 was used for all patients), the Brier score would be 0.175.

The number of deaths expected by the SAPS 3 score was 1322 (25.4%), resulting in an SMR of 0.91 (95% CI 0.86–0.96). The calibration curve was closer to the identity line than for the SAPS II score (Fig. 1). However, the mortality predicted by the SAPS 3 score was higher than the observed mortality for patients with a predicted risk between 0.50 and 0.90. The overestimation did not exceed 0.13. The Brier score was 0.131 (95% CI 0.126–0.136).

### Predictive value of individual items on miscalibration?

To determine if the miscalibration of the score was uniform or specific to certain score items, we compared the points attributed to each item according to the original work and the point weights derived from ELOISE data (Table 2). Items of the SAPS II score with a lowered association with mortality are extreme heart rate (<70 or >160 beats/minute), a Glasgow Coma Scale (GCS) score less than 6, a diagnosis of AIDS, a systolic blood pressure (SBP) less than 70 mm Hg and a serum sodium level less than 125 mmol/L. The SMR was 0.68 (95% CI 0.63-0.73) in patients with at least one of these items (*n* = 2230, 42.8%) and 0.89 (95% CI 0.81-0.97) in other patients.

**Table 2:** Reassessment of the points allocated to each item of Simplified Acute Physiology Score II items

Items of SAPS II score	Points^a^ (original/ELOISE study)	Difference
Age, years		
20–39	0/0	0
40–59	7/7	0
60–69	12/11	1
70–74	15/14	1
75–79	16/15	1
≥ 80	18/19	−1
Heart rate, beats/minute		
< 40	11/4	7
40–69	2/−5	7
70–119	0/0	0
120–159	4/3	1
≥ 160	7/−5	12
SBP, mmHg		
≥ 200	2/3	−1
100–199	0/0	0
70–99	5/3	2
< 70	13/7	6
PaO_2_, mmHg/FiO_2_		
No ventilation	0/0	0
≥ 200	6/3	3
100–199	9/6	3
< 100	11/11	0
Urinary output, L/day		
≥ 1.000	0/0	0
0.500–0.999	4/0	4
< 0.500	11/8	3
Serum urea level, mmol/L		
< 10.0	0/0	0
10.0–29.9	6/4	2
≥ 30.0	10/5	5
Body temperature		
< 39 °C	0/0	0
≥ 39 °C	3/−2	5
WBC count, ×10^3^/mm^3^		
< 1.0	12/8	4
1.0–19.9	0/0	0
≥ 20.0	3/2	1
Serum potassium, mmol/day		
≥ 3 and <5	0/0	0
< 3 or ≥5	3/2	1
Serum sodium level, mmol/L		
< 125	5/−1	6
≥ 125 and <145	0/0	0
≥ 145	1/5	−4
Serum bicarbonate level, mEq/L		
≥ 20	0/0	0
15–19	3/4	−1
< 15	6/9	−3
Bilirubin level, μmol/L		
< 68.4	0/0	0
68.4–102.5	4/2	2
≥ 102.6	9/10	−1
Glasgow Coma Scale score		
14–15	0/0	
11–13	5/5	0
9–10	7/9	−2
6–8	13/10	3
< 6	26/16	10
Chronic disease		
No	0/0	0
Metastatic cancer	9/8	1
Hematologic malignancy	10/9	1
AIDS	17/9	8
Type of admission		
Scheduled surgical	0/0	0
Medical	6/11	−5
Unscheduled surgical	8/9	−1

*Abbreviations: ELOISE* European Mortality & Length of Intensive Care Unit Stay Evaluation study, *FiO*_*2*_ Fractional inspired oxygen, *PaO*_*2*_ Partial pressure of arterial oxygen, *SAPS* Simplified Acute Physiology Score, *SBP* Systolic blood pressure, *WBC* White blood cell

^a^ Points proposed in the original SAPS II score and the points derived from the association between the items of the SAPS II score and the mortality reassessed with data from the ELOISE study

For the SAPS 3 score, items with a decreased association with mortality were the presence of metastatic cancer, intrahospital location before ICU admission, cardiac surgery, and a heart rate greater than 160 beats/minute (Additional file 3: Table S1). The SMRs were 0.82 (95% CI 0.76–0.88) in patients with at least one of these items (*n* = 2751 [52.8%]) and 1.10 (95% CI 1.00–1.21) in other patients.

### Reasons for admission to ICU and calibration

**Fig. 2:**
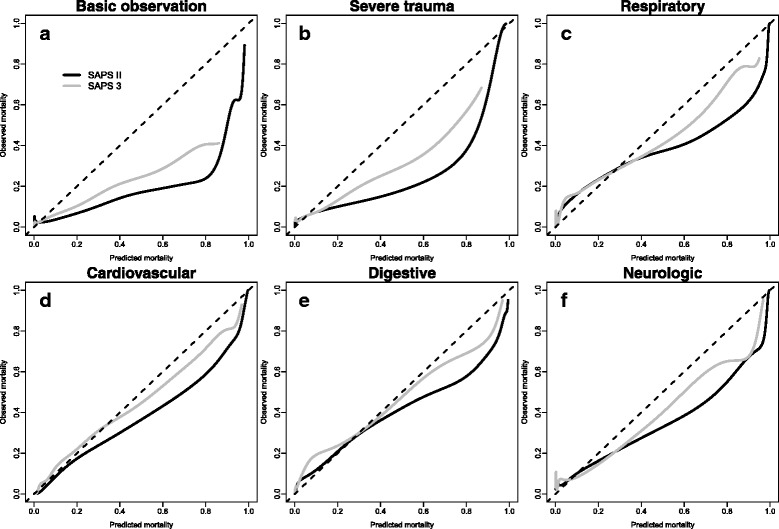
Calibration curves of the Simplified Acute Physiology Score (SAPS) II and SAPS 3 obtained by kernel function by reason for admission to the intensive care unit. **a** Basic observation. **b** Severe trauma. **c** Respiratory reason. **d** Cardiovascular reason. **e** Digestive reason. **f** Neurological reason

The calibration curves and the SMRs were assessed by reason for admission to ICU (Fig. 2 and Additional file 4: Table S2). For both SAPS scores, the overestimation of mortality was especially high in patients admitted to the ICU for a basic observation for SAPS II score (SMR 0.44, 95% CI 0.34–0.57) and for SAPS 3 score (SMR 0.68, 95% CI 0.52–0.88). In this subpopulation, the calibration curves deviated from the identity line even for low predicted risks. A similar but less marked trend was observed in patients admitted to the ICU for a severe trauma for SAPS II score (SMR 0.56, 95% CI 0.39–0.78) and for SAPS 3 score (SMR 0.73, 95% CI 0.51–1.02). For other reasons for admission, the miscalibration was less pronounced or even low. For instance, the SAPS 3 score was well calibrated in patients admitted to the ICU for a cardiovascular reason (SMR 0.94, 95% CI 0.86–1.03).

### Between-center variability

**Fig. 3:**
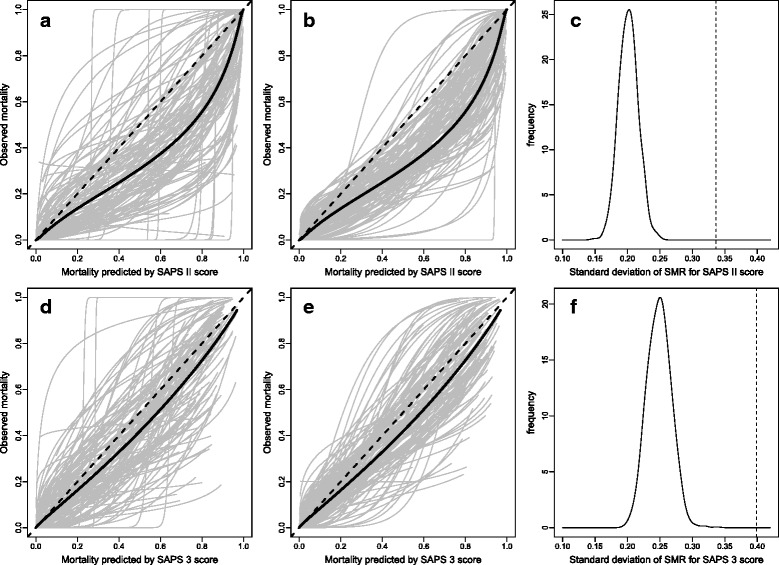
Observed and expected calibration curves for the Simplified Acute Physiology Score (SAPS) II score (*top*) and SAPS 3 score (*bottom*) in 120 centers and between-center variability in standardized mortality ratio (SMR). **a** Calibration curves of SAPS II in each of the 120 centers fitted with a logistic regression model. The *black line* represents the overall calibration curve. **b** Expected calibration curves of SAPS II under the assumption that the calibration is the same in all centers. The represented between-center variability is the random (sampling) variability. **c** Distribution of the SD of the center-specific SMRs under the assumption that the calibration of SAPS II is the same in all centers. The *vertical line* represents the observed value of the SD. **d**–**f** are the same figures shown in **a**–**c** repeated for the SAPS 3 score

We fitted a calibration curve of the SAPS II score separately in each of the 120 centers (Fig. 3a). The calibration curves varied considerably, but it was unclear if the variance was greater than what would be expected by chance alone. A typical pattern of calibration curves expected under the assumption that calibration is the same in all centers was obtained by randomly permuting the patients between centers (Fig. 3b). These figures suggest that the observed between-center variability in calibration is higher than the variability expected by chance. Figure 3c represents the distribution of the SD of the SMRs expected under the null hypothesis of absence of center effect on calibration. The observed SD of the SMR, represented by a vertical line, falls on the right-hand side of the distributions; the *p* value from the permutation test was less than 0.001. These findings show that the between-center variability in calibration of the SAPS II score is not well explained by random variability and suggest that center characteristics may add to this variability. The same findings were observed with the Brier score (Additional file 5: Figure S1A).

**Fig. 4:**
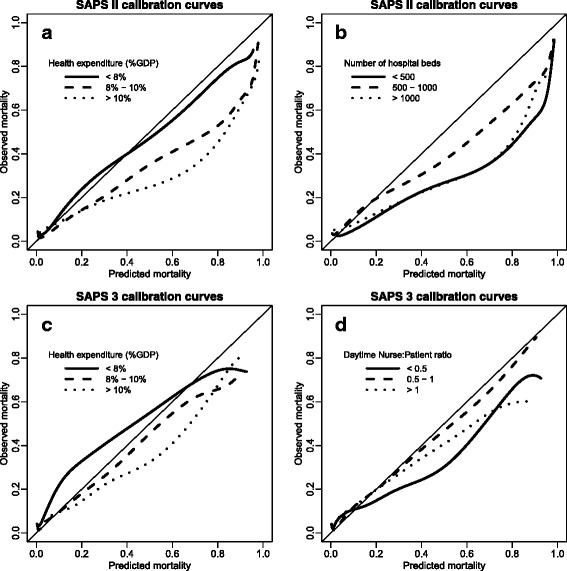
Calibration curves of the Simplified Acute Physiology Score (SAPS) II score obtained by kernel function according to (**a**) health expenditure expressed in percentage of gross domestic product (GDP) and (**b**) number of hospital beds, and calibration curves of the SAPS 3 score by (**c**) health expenditure expressed as a percentage of GDP and (**d**) daytime nurse/patient ratio

In regression models, the health expenditure and the number of hospital beds were significantly associated with the shape of the calibration curve of the SAPS II score (*p* < 0.001 and *p* = 0.004, respectively). The calibration curves according to these factors are shown in Fig. 4a and b. The SAPS II score was well calibrated in centers located in countries with health expenditures in 2012 less than 8% of the GDP, but the fit gets progressively worse as health expenditures grow. Furthermore, the overestimation of the risk of death was lower in ICUs in hospitals with 500–1000 beds than in centers in either smaller or larger hospitals. Other center characteristics were not significantly associated with the shape of the calibration curve (presence of IMCU *p* = 0.91, presence of an IMCU beds inside ICU *p* = 0.20, number of ICU adjusted beds *p* = 0.73, possibility of allocating extra beds inside the ICU *p* = 0.99, ICU nurse/patient ratio in daytime *p* = 0.10).

For SAPS 3, excess between-center variability in SMRs (Fig. 3d–f) (*p* < 0.001) and in Brier scores (Additional file 5: Figure S1B) was also found. In regression models, the health expenditure and ICU nurse/patient ratio in daytime were significantly associated with the shape of the calibration curve (*p* < 0.001 and *p* = 0.036, respectively). The corresponding calibration curves are shown in Fig. 4c and d. For centers located in countries with health expenditures less than 8% of the GDP, the SAPS 3 score underestimated the mortality. The other center characteristics were not significantly associated with the shape of the calibration curve (number of hospital beds *p* = 0.09, presence of IMCU *p* = 0.68, presence of an IMCU beds inside ICU *p* = 0.80, number of ICU adjusted beds *p* = 0.62, possibility of allocating extra beds inside the ICU *p* = 0.96). Data for SMR and Brier score by level of health expenditure are shown in Additional file 6 (Table S3) for both SAPS scores.

## Discussion

The SAPS II and SAPS 3 scores globally overestimated mortality, with an overestimation more marked for the SAPS II (SMR 0.75) than for the SAPS 3 (SMR 0.91). Although overestimation of mortality has been reported by others [10, 19–22], we show that this miscalibration does not affect all patients and all ICUs similarly. First, the miscalibration depended on the level of the predicted risk in each patient and on the specific items of the scores presented by the patients. Second, the calibration varied across centers; the miscalibration was more important in countries with high health expenditures, as well as in small and large hospitals than in hospitals of medium size.

The scores calibrated well when the predicted risk was low (below a predicted risk of 0.30 approximately), and the overestimation increased up to 0.25 for the SAPS II score (0.13 for the SAPS 3) at around 0.75 predicted mortality. The points originally attributed to some items of the score do not capture correctly the increase of mortality anymore, owing to the magnitude of the associations changed since the development of the score. The main items of predictive scores with a lowered association were heart rate, GCS (<6), and chronic disease (AIDS) for the SAPS II score and anatomical site of surgery (transplantation, trauma–other), intrahospital location before ICU admission, comorbidities (metastatic cancer), heart rate (≥160 beats/minute) for the SAPS 3 score. Some of these decreased associations (heart rate, SBP) may be explained by modern automatic or semiautomatic data collection methods that have been shown to find more “pathological” elements, thereby inflating the assigned SAPS scores [23]. The decreased association of AIDS may be explained by the introduction of highly effective therapies against HIV. The decreased predictive capacity of GCS for SAPS II may be caused by a common misconception about the evaluation rules [24]: A sedated patient is sometimes mistakenly attributed the worst score (3), whereas the score should reflect the state in which we believe the patients would be without sedation. Another possible explanation is that data are of lower quality in real life than in research validation studies, and random errors would also dilute the associations.

The calibration of the scores varied across the reason for admission to ICU. Especially, the mortality predicted by SAPS II and SAPS 3 scores was too high when the scores were applied to patients admitted to the ICU for a basic observation or for a severe trauma. Possibly, the relationship between the mortality and biological parameters involved in the predictive scores is different in patients admitted to the ICU for any traumatic injuries responsible for a strong physiological stress reaction and in patients admitted for another reason. The biological values may capture well the stage of medical diseases but poorly the effects of the homeostatic mechanisms favoring recovery after trauma. In addition to the influence of characteristics of patients on calibration, we detected a large heterogeneity across centers. The variability of the calibration was too large to be explained only by random sampling. Some characteristics of centers were associated with the miscalibration of the SAPS scores: the country’s health expenditure (SAPS II and SAPS 3), number of hospital beds (SAPS II), and the daytime nurse/patient ratio (SAPS 3). If we have no reasonable explanation for the variation by hospital size, the effect of health expenditure may be explained by the amount of resources available in the ICU to treat patients. In low-expenditure countries, lifesaving medical technologies may be underused or rationed, which may cause higher mortality more comparable to mortality rates that existed 25 years ago, when the SAPS II score was developed. Any new effective medical treatment is bound to reduce the predictive value of the medical condition it treats; for example, survival after a myocardial infarction has improved since the introduction of percutaneous transluminal coronary angioplasty and thrombolytic therapies.

This study has several limitations. Analyzed data were collected as part of the ELOISE study, in which researchers sought to detect an effect on mortality of the presence of an IMCU in the hospital. Because the ELOISE study was not designed to assess the determinants of the calibration of the SAPS II and SAPS 3 mortality scores, some determinants of the calibration were not collected, such as the policy for end-of-life care. Moreover, ICUs participated on a voluntary basis, and they may not represent all European ICUs.

## Conclusions

This study suggests that the prognostic significance of SAPS II and SAPS 3 scores is not uniform across Europe, because it depends on both patient-specific and center-specific characteristics. Another important part of variability remains unexplained. This suggests that users of these scores should proceed with caution, especially if ICUs that serve different patient populations and that are located in countries with different levels of health expenditures are being compared. More generally, our results suggest that the external validity of prognostic scores developed in a given context should not be taken for granted, as well as that local revalidation is a useful precaution. Furthermore, it may be prudent to reassess periodically the predictive capacity of even well-established scores because changes in medical treatments may alter the value of such instruments.

## Additional files

Additional file 1: Additional details on data collection. (DOCX 14 kb)

Additional file 2: Additional details on statistical methods. (DOCX 16 kb)

Additional file 3: Original and reassessed points of the items of SAPS 3 score. (DOCX 18 kb)

Additional file 4: SMRs and Brier scores of the SAPS II and SAPS 3 scores, by reason for admission to ICU. (DOCX 11 kb)

Additional file 5: Distribution of the SD of the center-specific Brier scores under the assumption that the calibration is the same in all centers for (**a**) the SAPS II score and (**b**) the SAPS 3 score. The *vertical lines* represent the observed SD of Brier score. (DOCX 24 kb)

Additional file 6: SMRs and Brier scores of the SAPS II and SAPS 3 scores, by categories of health expenditure (percentage of GDP). (DOCX 12 kb)

Additional file 7: Ethics committees. (DOCX 33 kb)

## Abbreviations

ELOISE: European Mortality & Length of Intensive Care Unit Stay Evaluation study
FiO_2_: Fractional inspired oxygen
GCS: Glasgow Coma Scale
GDP: Gross domestic product
ICU: Intensive care unit
IMCU: Intermediate care unit
PaO_2_: Partial pressure of arterial oxygen
SAPS: Simplified Acute Physiology Score
SBP: Systolic blood pressure
SMR: Standardized mortality ratio
WBC: White blood cell
