# Supplementary material for: Determinants of the calibration of SAPS II and SAPS 3 mortality scores in intensive care: a European multicenter study
Source: Crit Care. 2017 Apr 4;21:85. doi: 10.1186/s13054-017-1673-6 (PMC5379500; doi:10.1186/s13054-017-1673-6)
Supplement: Supplementary file 1 — Additional details on data collection. (DOCX 14 kb) [file 13054_2017_1673_MOESM1_ESM.docx]

**Data collection**

Each ICU coordinator answered the Study unit questionnaire to provide information about the hospital and the organization of the study unit (ICU) via web (through the software managed by the European Society of Intensive Care Medicine). Clear definition of each item was given in the study protocol, before answering the questionnaire. All ICU coordinators were members of the staff of the study ICU (mainly medical doctors, some nurses) and downloaded from the website (<http://eloise.esicm.org>):

- a paper form to collect all the variables required for the study, including those to obtain severity scores, and a form with clear definition of each item. Each variable required to calculate each score was presented with the ranges reported in the original paper. For instance SAPS3 Heat rate was showed as follows:  <120, ≥120  and < 160, ≥160;
- an excel file designed for recording data collected, filled in by the ICU coordinator and then sent to the Coordination and Communication Centre (CCC).

Most of ICU coordinators used paper forms, and then filled in excel file. We did not investigate the presence of patient data management systems in the ICUs. The SAPS II and SAPS 3 scores were computed electronically on the excel file software, after completing data collection.

At the end of the study patient data collection, each study unit was required to re-abstract the data of a maximum of three cases identified by the CCC for quality control. Data quality control was performed on 281 (4%) records. The median number of missing data was 0.29 (IQR 0.11 to 0.62) per unit. Data quality was excellent, as most reliability coefficients exceeded 0.85 for data involved in the assessment of the calibration of SAPS II and SAPS 3 scores [1].

The following reasons for admission to ICU were asked in the questionnaire:

- cardiovascular reason: cardiac arrest, hypovolemic shock, septic shock, cardiogenic shock, anaphylactic (mixed and undefined) hock, chest pain (with ECG changes), hypertensive crisis, rhythm disturbances, cardiac failure without shock, other cardiovascular reason
- digestive reason: bleeding, acute abdomen, severe pancreatis, other digestive reason
- neurological reason: coma, stupor, obtunded, vigilance disturbances, confusion, delirium, seizures, focal neurological deficit, intracranial mass effect, other neurological reason
- respiratory reason: acute lung injury and acute respiratory distress syndrome, acute respiratory failure on chronic pulmonary disease, other respiratory reason
- other reason: severe trauma or other

Basic observation was defined as follow: no other reason for admission, a maximum score of 2 for the item “Respiratory” of SOFA score and a maximum score of 1 (mild dysfunction) for the following five SOFA items: “Neurology”, “Cardiovascular”, “Liver”, “Coagulation”, “Renal creatinin”.

**Bibliography**

[1] Capuzzo M, Volta C, Tassinati T, Moreno R, Valentin A, Guidet B, Iapichino G, Martin C, Perneger T, Combescure C et al. Hospital mortality of adults admitted to Intensive Care Units in hospitals with and without Intermediate Care Units: a multicentre European cohort study. Crit Care. 2014 ; 18:551.
