# Supplementary material for: Determinants of the calibration of SAPS II and SAPS 3 mortality scores in intensive care: a European multicenter study
Source: Crit Care. 2017 Apr 4;21:85. doi: 10.1186/s13054-017-1673-6 (PMC5379500; doi:10.1186/s13054-017-1673-6)
Supplement: Supplementary file 2 — Additional details on statistical methods. (DOCX 16 kb) [file 13054_2017_1673_MOESM2_ESM.docx]

**Statistical methods**

**Assessment of calibration of SAPS II and 3 scores**

The calibration curves of the SAPS II and SAPS 3 scores for the prediction of in hospital death were assessed. These curves show the relationship between the observed mortality and the mortality predicted by the scores. The observed risk function of the predicted mortality was assessed using smooth kernel functions [[1](#_ENREF_1)]. Then, the observed mortality was plotted against the predicted mortality. The identity line represents a perfect calibration of the score. If the curve is below (above) the identity line, the score overestimates (underestimates) the mortality. Stronger is deviation from the identity line, greater is the miscalibration. To obtain smooth free-distribution calibration curves, we assessed the observed mortality according to the predicted mortality using a normal kernel function [[1](#_ENREF_1)]. Additionally, we calculated the Brier score and the Standardized Mortality Ratio (SMR). The former is the mean square difference between the mortality predicted by the score and the actual binary outcome [[2](#_ENREF_2)]. The Brier score captures the calibration and the discrimination power of the predictive score. To facilitate its interpretation, we also assessed a reference value equal to the Brier score when the predicted mortality is assumed the same for all patients and equal to the overall mortality. The SMR is the ratio of observed count of death to count of death expected according to the mortality score. A SMR greater (resp. lower) than one indicates an underestimation (resp. overestimation) of the mortality by the predictive score. Ninety-five percent confidence intervals were estimated using the method proposed by Redelmeier et al for Brier score [[3](#_ENREF_3)] and Byar’s approximation for SMR [[4](#_ENREF_4)].

**Calibration and centers’ characteristics**

The variance of the set of center-specific Brier scores and SMRs were indices of the between-centers variability in calibration. We used a permutation test to determine if these indices were compatible with the hypothesis that the calibration was the same in all centers: 1. we randomly permuted patients between centers, maintaining the initial sample size for each center, and estimated a calibration curve for each center and obtained the center-specific SMR and Brier score; 2. we calculated the standard deviation over the 120 centers of (a) the SMR (b) the Brier score; 3. we repeated this procedure 1000 times to obtain the distribution of the variance of the calibration indices under random variation only; 4. we compared the observed variability with the expected variability under random variation. If the observed standard deviation fell above the 97.5^th^ percentile of the standard deviations obtained under randomness, it would signal statistically significant between-center heterogeneity in calibration. Because of the limited sample size per center, assessment of calibration curves with kernel functions was not appropriate. We fitted the calibration curves according to the method proposed by Finazzi et al [[5](#_ENREF_5)]. Briefly, the principle of this approach is to model the logit of the observed risk of death by the logit of the mortality predicted by the SAPS II score with a logistic regression model.

To evaluate if center characteristics have an effect on the calibration of the SAPS II score, we modeled the calibration curve using the approach proposed by Finazzi et al and we introduced interaction terms between center characteristic and the logit of the predicted mortality. The models with and without interaction terms were compared with likelihood ratio tests. A statistically significant difference between model means that the parameters of the calibration curve vary with center characteristic. Since these analyses were conducted on the whole sample, the sample size was large enough to use polynomial functions of the logit of the predicted mortality. Only terms of degree 1 and 3 were retained in the models because of their statistical significance. Generalized estimating equations were used to account for the clustering.

**Bibliography**

1. Copas JB. Plotting p against x. Journal of the Royal Statistical Society Series C (Applied Statistics). 1983; 32:25-31.

2. Brier GW. Verification of forecasts expressed in terms of probability. Mon Weather Rev. 1950; 78:1-3.

3. Redelmeier DA, Bloch DA, Hickam DH. Assessing predictive accuracy: how to compare Brier scores. J Clin Epidemiol. 1991; 44:1141-6.

4. Breslow NE, Day NE. Statistical methods in cancer research. Volume II--The design and analysis of cohort studies. IARC Sci Publ; 1987.

5. Finazzi S, Poole D, Luciani D, Cogo PE, Bertolini G. Calibration belt for quality-of-care assessment based on dichotomous outcomes. PLoS One. 2011; 6:16110.
