# Supplementary material for: Determinants of the calibration of SAPS II and SAPS 3 mortality scores in intensive care: a European multicenter study
Source: Crit Care. 2017 Apr 4;21:85. doi: 10.1186/s13054-017-1673-6 (PMC5379500; doi:10.1186/s13054-017-1673-6)
Supplement: Supplementary file 3 — Original and reassessed points of the items of SAPS 3 score. (DOCX 18 kb) [file 13054_2017_1673_MOESM3_ESM.docx]

**Table S1.** Original and re-assessed points of the items of SAPS 3 score. The re-assessed points were ten times the regression coefficients from a logistic regression model to predict mortality with ELOISE dataset..

| Items of SAPS 3 | **Studies (Original/ELOISE)** | **Difference** |
| --- | --- | --- |
| Age (years) |  |  |
| 20-39 | 0/0 | 0 |
| 40-59 | 5/5 | 0 |
| 60-69 | 9/10 | -1 |
| 70-74 | 13/13 | 0 |
| 75-79 | 15/15 | 0 |
| ≥80 | 18/19 | -1 |
| Comorbidities |  |  |
| No comorbidities | 0/0 | 0 |
| Cancer therapy | 3/3 | 0 |
| Chron, HF (NYHA IV), Haematological cancer | 6/6 | 0 |
| Cirrhosis, AIDS | 8/6 | 2 |
| Metastatic cancer | 11/5 | 6 |
| Length of Stay before ICU admission (days) |  |  |
| <14 | 0/0 | 0 |
| 14-27 | 6/6 | 0 |
| ≥28 | 7/9 | -2 |
| Intra-hospital location before ICU admission |  |  |
| No intra-hospitalization location |  |  |
| Emergency room | 5/-2 | 7 |
| Other ICU | 7/2 | 5 |
| Other | 8/0 | 8 |
| Use of major therapeutic options before ICU admission |  |  |
| No vasoactive drugs | 0/0 | 0 |
| Vasoactive drugs | 3/2 | 1 |
| ICU admission |  |  |
| Planned | 0/0 | 0 |
| Unplanned | 3/3 | 0 |
| Reason for ICU admission* |  |  |
| ICU admission* | 16/16 | 0 |
| All others | 0/0 | 0 |
| Cardiovascular: Rhythm disturbances** | -5/-2 | -3 |
| Cardiovascular: Shock (septic, anaphylactic,…) | 5/2 | 3 |
| Digestive: Acute abdomen, Other | 3/6 | -3 |
| Digestive: Severe pancreatitis | 9/5 | 4 |
| Hepatic : Liver failure | 6/1 | 5 |
| Neurologic : Seizures** | -4/-6 | 2 |
| Neurologic : Coma, confusion, delirium ... | 4/3 | 1 |
| Neurologic : Focal neurologic deficit | 7/3 | 4 |
| Neurologic : Intracranial mass effect | 10/9 | 1 |
| Anatomical site of surgery |  |  |
| All others | 0/0 | 0 |
| Transplantation surgery | -11/-4 | -7 |
| Trauma – other, isolated | -8/1 | -9 |
| Cardiac surgery | -6/-17 | 11 |
| Neurosurgery | 5/1 | 4 |
| Surgical status |  |  |
| Scheduled surgery | 0/0 | 0 |
| No surgery | 5/11 | -6 |
| Emergency surgery | 6/8 | -2 |
| Acute infection at ICU admission |  |  |
| No acute infection | 0/0 | 0 |
| Nosocomial | 4/4 | 0 |
| Respiratory | 5/5 | 0 |
| Estimated Glasgow Coma Scale (lowest) |  |  |
| ≥13 | 0/0 | 0 |
| 7 - 12 | 2/4 | -2 |
| 6 | 7/5 | 2 |
| 5 | 10/12 | -2 |
| 3 - 4 | 15/12 | 3 |
| Total bilirubin level (highest), mg/dL |  |  |
| <2 | 0/0 | 0 |
| 2 - 5.9 | 4/2 | 2 |
| ≥6 | 5/12 | -7 |
| Body temperature (°C) |  |  |
| ≥35 | 0/0 | 0 |
| <35 | 7/3 | 4 |
| Creatinine (highest), mg/dL |  |  |
| <1.2 | 0/0 | 0 |
| 1.2 - 1.9 | 2/4 | -2 |
| 2 - 3.4 | 7/10 | -3 |
| ≥3.5 | 8/6 | 2 |
| Heart rate (highest), beats/minute |  |  |
| <120 | 0/0 | 0 |
| 120-159 | 5/4 | 1 |
| ≥160 | 7/1 | 6 |
| Leukocytes (highest), G/L |  |  |
| <15 | 0/0 | 0 |
| ≥15 | 2/2 | 0 |
| Hydrogen ion concentration (lowest), pH |  |  |
| >7.25 | 0/0 | 0 |
| ≤7.25 | 3/6 | -3 |
| Platelets (lowest), G/L |  |  |
| ≥100 | 0/0 | 0 |
| 50-99 | 5/3 | 2 |
| 20-49 | 8/6 | 2 |
| <20 | 13/11 | 2 |
| SBP (lowest) (mm Hg) |  |  |
| ≥120 | 0/0 | 0 |
| 70-119 | 3/4 | -1 |
| 40-79 | 8/8 | 0 |
| <40 | 11/15 | -4 |
| Oxygenation |  |  |
| PaO2≥60 and no MV | 0/0 | 0 |
| PaO2<60 and no MV | 5/5 | 0 |
| PaO2/FiO2 ≥ 100 and MV | 7/2 | 5 |
| PaO2/FiO2 < 100 and MV | 11/10 | 1 |

*Every patient gets an offset of 16 points for being admitted (to avoid negative SAPS 3 Scores).

** If both reasons for admission are present, only the worse value (-4) is scored.
