# Supplementary material for: Determinants of the calibration of SAPS II and SAPS 3 mortality scores in intensive care: a European multicenter study
Source: Crit Care. 2017 Apr 4;21:85. doi: 10.1186/s13054-017-1673-6 (PMC5379500; doi:10.1186/s13054-017-1673-6)
Supplement: Supplementary file 4 — SMRs and Brier scores of the SAPS II and SAPS 3 scores, by reason for admission to ICU. (DOCX 11 kb) [file 13054_2017_1673_MOESM4_ESM.docx]

**Table S2.** SMRs and Brier scores of the SAPS II and SAPS 3 scores by reason for admission to ICU. Each SMR and Brier score is reported with the 95% confidence interval and the sample size (n).

|  | SAPS II | SAPS 3 |
| --- | --- | --- |
| **SMRs (95%CI)** |  |  |
| Reason for admission |  |  |
| Cardiovascular | 0.79 (0.72 to 0.87), n=1243 | 0.94 (0.86 to 1.03), n=1245 |
| Digestive | 0.89 (0.76 to 1.04), n=520 | 1.09 (0.92 to 1.27), n=519 |
| Neurological | 0.74 (0.65 to 0.85), n=785 | 0.83 (0.72 to 0.94), n=790 |
| Respiratory | 0.89 (0.78 to 1.00), n=963 | 0.99 (0.88 to 1.12), n=971 |
| Severe trauma | 0.56 (0.39 to 0.78), n=252 | 0.73 (0.51 to 1.02), n=254 |
| Basic observation | 0.44 (0.34 to 0.57), n=1103 | 0.68 (0.52 to 0.88), n=1087 |
