# Supplementary material for: Determinants of the calibration of SAPS II and SAPS 3 mortality scores in intensive care: a European multicenter study
Source: Crit Care. 2017 Apr 4;21:85. doi: 10.1186/s13054-017-1673-6 (PMC5379500; doi:10.1186/s13054-017-1673-6)

**Fig S1.** Distribution of the standard deviation of the center-specific Brier scores under the assumption that the calibration is the same in all centers for A) the SAPS II score and B) the SAPS 3 score. The vertical lines represent the observed standard deviation of Brier score. Under the assumption that the calibration is the same in all centers, the standard deviation of Brier score would fall between 0.03 and 0.05 approximately. Since the observed standard deviation is much greater than 0.05, the variability of Brier score across centers is not well explained by the sampling variability.


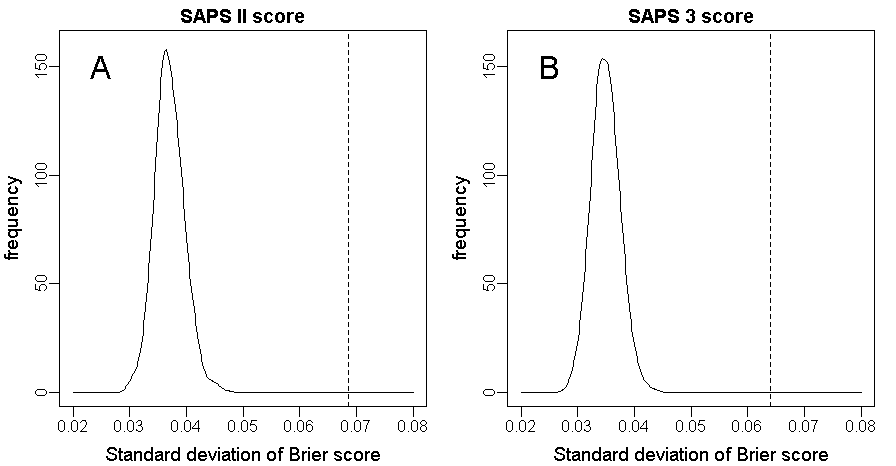

Supplement: Supplementary file 5 — Distribution of the SD of the center-specific Brier scores under the assumption that the calibration is the same in all centers for (a) the SAPS II score and (b) the SAPS 3 score. The vertical lines represent the observed SD of Brier score. (DOCX 24 kb) [file 13054_2017_1673_MOESM5_ESM.docx]
