# Supplementary material for: Determinants of the calibration of SAPS II and SAPS 3 mortality scores in intensive care: a European multicenter study
Source: Crit Care. 2017 Apr 4;21:85. doi: 10.1186/s13054-017-1673-6 (PMC5379500; doi:10.1186/s13054-017-1673-6)
Supplement: Supplementary file 6 — SMRs and Brier scores of the SAPS II and SAPS 3 scores, by categories of health expenditure (percentage of GDP). (DOCX 12 kb) [file 13054_2017_1673_MOESM6_ESM.docx]

**Table S3.** SMRs and Brier scores of the SAPS II and SAPS 3 scores by categories of health expenditure (percentage of GDP). Each SMR and Brier score is reported with the 95% confidence interval and the sample size (n).

|  | SAPS II | SAPS 3 |
| --- | --- | --- |
| **SMRs** |  |  |
| Health expenditure (%GDP) |  |  |
| <8% | 1.00 (0.89 to 1.12), n=936 | 1.19 (1.06 to 1.34), n=952 |
| 8% – 10% | 0.72 (0.66 to 0.80), n=2096 | 0.92 (0.84 to 1.01), n=2078 |
| >10% | 0.66 (0.60 to 0.73), n=2177 | 0.78 (0.71 to 0.85), n=2176 |
| All | 0.75 (0.71 to 0.79), n=5209 | 0.91 (0.86 to 0.96), n=5206 |
| **Brier scores** |  |  |
| Health expenditure (%GDP) |  |  |
| <8% | 0.121 (0.109 to 0.133), n=936 | 0.153 (0.141 to 0.164), n=952 |
| 8% – 10% | 0.124 (0.115 to 0.132), n=2096 | 0.120 (0.112 to 0.128), n=2078 |
| >10% | 0.145 (0.137 to 0.153), n=2177 | 0.132 (0.124 to 0.140), n=2176 |
| All | 0.132 (0.127 to 0.137), n=5209 | 0.131 (0.126 to 0.136), n=5206 |
