# Supplementary material for: Determinants of the calibration of SAPS II and SAPS 3 mortality scores in intensive care: a European multicenter study
Source: Crit Care. 2017 Apr 4;21:85. doi: 10.1186/s13054-017-1673-6 (PMC5379500; doi:10.1186/s13054-017-1673-6)
Supplement: Supplementary file 7 — Ethics committees. (DOCX 33 kb) [file 13054_2017_1673_MOESM7_ESM.docx]

**Determinants of the calibration of SAPS II and SAPS 3 mortality scores in intensive care: a European multi-center study**

Poncet A., Perneger T.V., Merlani P., Capuzzo M., Combescure C.

**Ethical approval:**

Ethics requirements differed by country. Given the design of Eloise study, and given the regulation in Austria, Poland and Switzerland no Ethics approval was required. In France, the “Groupe Ethique de l’association pour la formation et la Recherche en anesthésie-réanimation” approved the study. In the UK, the National Research Ethics Committee London Harrow approved the study. In some countries (Belgium, Denmark, and Norway), the ethical approval obtained by the coordinating centre was valid for all the centres in the same country. In some countries (Ireland, Italy), ethics requirements differed by centres of the same country. Moreover, in some centres the study was considered and managed as an audit. However, each unit was responsible for obtaining local permissions, as necessary, according to local regulations.

The following ethical bodies approved the study: Commissie voor Medische Ethiek - Ghent University Hospital; Comité d’éthique des Cliniques de l’Europe; Comité d'Ethique Hospitalo-Facultaire Universitaire de Liège; Ethisch Comité O.L. Vrouwziekenhuis Aalst; Ethics Committe of Teaching Hospital and Medical Faculty Plzen; Etická komise FN Brno; Ethics Committee of University Hospital in Hradec Kralove; Ethics Committe of Region Southern Denmark; Ethics Committee of the University of Leipzig, Germany; Ethik-Kommission der Medizinischen Fakultät der Ruhr Universität Bochum, Germany; Scientific Committee of Attikon University Hospital; Scientific board of General Hospital of G.Gennimatas, Thessaloniki; Scientific Board of University General Hospital of Thessaloniki AHEPA; Scientific Committee of Aretaieion University Hospital, Athens; Educational-Ethics Committee of the University Hospital of Larissa; University hospital of Ioannina Ethics Committee; Scientific Council of Hippokration General Hospital of Thessaloniki; Sotiria Hospital Ethics Committee, Athens; Ethics Committe of Papanikolaou Hospital , Thessaloniki; Scientific Committee at "Agioi Anargyroi" Hospital, Athens; Naval Hospital of Athens Ethics Committee; Scientific Board of Sismanoglio General Hospital; Scientific committee of Center IASO Thessalias; Scientific Committee of Artas General Hospital; Clinical Research Ethics Committee of the Cork Teaching Hospitals; Ethics (Medical Research) Committee, Beaumont Hospital, Dublin; Ethics and Medical Research Committee, St Vincent's Health Care Group Ltd.; Comitato Etico indipendente dell'Azienda Ospedaliero-Universitaria di Bologna; Comitato Etico della Provincia di Ferrara; Comitato etico interaziendale AUSL Bologna e Imola; Comitato bioetico dell'ARNAS Ospedale Civico Di Cristina Benfratelli di Palermo; Modena Local Ethical committee; Comitato Etico Azienda Ospedaliera San Paolo, Milano; Ethics committee of REK Sør-Øst. Centre: Stavanger University Hospital; REK Sør-Øst. Centre: Ålesund Hospital; Comissão de Ética para a Saúde do CHLC; Comissão de Ética para a Saúde do Centro Hospitalar de Coimbra; Unidade Local de Saúde de Matosinhos Ethics Committee; Comissão de ética da Unidade Local de Saúde do Alto Minho; Comissão de Ética para a Saúde do Hospital S. João; Comissão de Ética para a Saúde do Centro Hospitalar de Setúbal; Ethics Committee of Emergency County Hospital Cluj Napoca; Ethics Committee of Emergency Institute of Cardiovascular Diseases "Prof.Dr C.C.Iliescu", Bucharest, Romania; University Emergency County Hospital Mures Local Ethical Committte; Comisia Locala de Etica - Spitalul Universitar de Urgenta Elias; Ethics Committee of Emergency Institute of Cardiovascular Diseases "Prof.Dr. C.C.Iliescu", Bucharest, Romania; Clinical Emergency Hospital of Bucharest Local Ethical Committte; Ethics Committee of Clincal Emergency County Timisoara; Education and Medical research Comittee of Spitalul Judetean de Urgenta “Dr. Constantin Opris” Baia Mare; Consiliul Etical Institutului Clinic Fundeni Center; Comité Ético de Investigación Clínica de Cartagena; Investigation Committee of Hospital Universitario de Torrejón; Comité de Etica de Investigación Clínica de la Universidad de Navarra; Istanbul University Cerrahpasa Medical School, Clinical Research Ethics Committe; Ethics Committee of the Ankara Numune Training and Research Hospital; Clinical Researches Etics Committee of Tepecik Training and Research Hospital; Mersin University Clinical Research Ethics Committee; Bakırköy Dr. Sadi Konuk Education and Searching Hospital.
